# Mesenchymal stem cells: Emerging concepts and recent advances in their roles in organismal homeostasis and therapy

**DOI:** 10.3389/fcimb.2023.1131218

**Published:** 2023-03-09

**Authors:** Peisheng Liu, Yongqian An, Ting Zhu, Siyuan Tang, Xiaoyao Huang, Shijie Li, Fei Fu, Ji Chen, Kun Xuan

**Affiliations:** ^1^ The College of Life Science, Northwest University, Xi’an, Shaanxi, China; ^2^ State Key Laboratory of Military Stomatology & National Clinical Research Center for Oral Diseases & Shaanxi International Joint Research Center for Oral Diseases, Center for Tissue Engineering, The Fourth Military Medical University, Xi’an, Shaanxi, China; ^3^ Department of Preventive Dentistry, School of Stomatology, The Fourth Military Medical University, Xi’an, Shaanxi, China; ^4^ Department of Stomatology, 962 Hospital of People's Liberation Army of China, Harbin, Heilongjiang, China; ^5^ School of Basic Medicine, The Fourth Military Medical University, Xi’an, Shaanxi, China; ^6^ Department of Oral Implantology, School of Stomatology, The Fourth Military Medical University, Xi’an, Shaanxi, China

**Keywords:** mesenchymal stem cells, cell therapy, cell-host interplay, cell release, microenvironment

## Abstract

Stem cells play a crucial role in re-establishing homeostasis in the body, and the search for mechanisms by which they interact with the host to exert their therapeutic effects remains a key question currently being addressed. Considering their significant regenerative/therapeutic potential, research on mesenchymal stem cells (MSCs) has experienced an unprecedented advance in recent years, becoming the focus of extensive works worldwide to develop cell-based approaches for a variety of diseases. Initial evidence for the effectiveness of MSCs therapy comes from the restoration of dynamic microenvironmental homeostasis and endogenous stem cell function in recipient tissues by systemically delivered MSCs. The specific mechanisms by which the effects are exerted remain to be investigated in depth. Importantly, the profound cell-host interplay leaves persistent therapeutic benefits that remain detectable long after the disappearance of transplanted MSCs. In this review, we summarize recent advances on the role of MSCs in multiple disease models, provide insights into the mechanisms by which MSCs interact with endogenous stem cells to exert therapeutic effects, and refine the interconnections between MSCs and cells fused to damaged sites or differentiated into functional cells early in therapy.

## 1 Introduction

Mesenchymal stem cells (MSCs) are the current focus of extensive works worldwide, directed to elucidate their nature and properties, as well as to develop cell-based therapies for various diseases (Kfoury and Scadden, 2015; Harrell et al., 2019; Lerman, 2021; Zhu et al., 2021; Hoang et al., 2022). Our understanding of the therapeutic potential of MSCs has been promoted by research progresses such as the identification and characterization of MSCs from diverse origins (Darzi et al., 2016; Donders et al., 2016; Lu et al., 2016; Cooper et al., 2020; Medrano-Trochez et al., 2021), recognition of MSC contributions to organismal homeostasis and diseases (Sui B. D et al., 2016; Neri and Borzì, 2020; Sui et al., 2020; Krampera and Le Blanc, 2021; Spallanzani, 2021), the application or intervention of MSCs in tissue engineering and cytotherapy (Akiyama et al., 2012; Cassandras et al., 2020; McNeill et al., 2020), and clarification of transcription factors and signaling pathways capable of controlling the behaviors of MSCs (Feng et al., 2017; Elbaz et al., 2019; Zecchini et al., 2019; Choi et al., 2021).

Further illuminating matters, function and therapeutic efficacy of MSCs are highly regulated by the surrounding niche/microenvironment (Zhu et al., 2016; Mehrbani Azar et al., 2018; Tejero et al., 2019; Gilchrist et al., 2021), and studies on skeletal degenerative and autoimmune conditions have highlighted the essence of cell-host interplay in the forms of cell-cell contact and paracrine secretion in MSC cytotherapy (Liu S. et al., 2015; Kou et al., 2018; Li et al., 2018; Liu et al., 2018; Tejero et al., 2019; Ha et al., 2020). Interestingly, these interactions provide persistent therapeutic benefits that remain detectable long after the disappearance of transplanted MSCs (Liu S. et al., 2015; Ng et al., 2015). Therefore, there is an urgent need for a more complete understanding of the molecular mechanisms and biological processes underlying MSC therapies.

In this review, we summarize recent developments regarding the role of MSCs in a variety of disease models and provide insight into the mechanisms by which MSCs interact with endogenous stem cells to exert therapeutic effects, refining the interconnection between MSCs and cells fused or differentiated into functional cells at the site of damage in the early stages of treatment. This landscape offers a unifying explanation of how the MSC therapy re-establishes the health of the diseased organism across diverse tissues with long-lasting beneficial profiles, shedding light on the future development of cell-free and cell-targeted therapies.

## 2 The MSC overview: Potent candidates in cytotherapy

**Figure 1:**
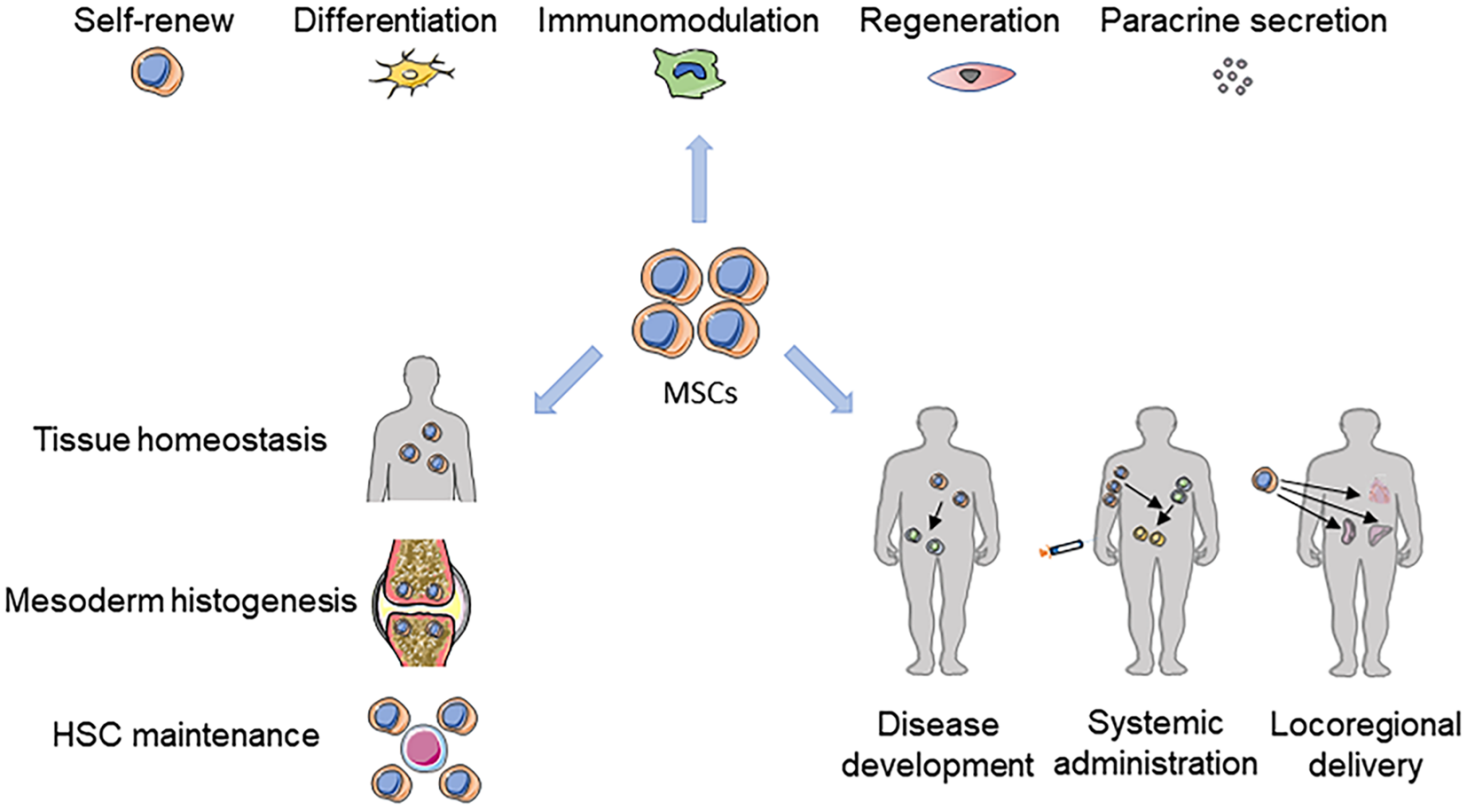
The MSC landscape in tissue homeostasis, disease and therapies. The powerful self-renewal and differentiation capabilities, immunomodulatory capacity, regeneration-promoting functions, and paracrine effects of MSCs all play important roles in tissue homeostasis, mesodermal histogenesis, and maintenance of HSC function. MSCs can intervene in disease development and treat related diseases through systemic or local applications.

The concept of MSCs originated from seminal studies performed by Friedenstein et al. who confirmed that postnatal mammalian bone marrow (BM) contains a subset of non-hematopoietic stromal cells that are both self-renewing and multipotent. Currently, the MSC concept is referred to as primitive cells capable of adherence, forming fibroblastic colonies and multilineage differentiation when cultured *ex vivo* (Kfoury and Scadden, 2015). MSCs, other than those derived from the BM (BMMSCs) (Jiang et al., 2002), have been shown to reside in a variety of tissues, such as the adipose (ADSCs) (Zuk et al., 2002), umbilical cord (UCMSCs) (Erices et al., 2000), tendon (TSPCs) (Bi et al., 2007), dental pulp (DPSCs) (Gronthos et al., 2000), periodontal ligament (PDLSCs) (Seo et al., 2004) and even the exfoliated deciduous teeth (SHED) (Miura et al., 2003). The surface profiles of MSCs are still not fully understood; we use a complex of heterogeneous distinct subsets of MSCs, which can be considered as a network of stromal components with interrelated and complementary *in vivo* capabilities in the maintenance of tissue homeostasis (Kfoury and Scadden, 2015) (**Figure 1**). Despite recent studies on identifying functional heterogeneity and specific markers of these cells (Chan et al., 2015; Worthley et al., 2015), the ready abilities of isolation, amplification and differentiation have made MSCs an ideal subject for extensive investigation in tissue engineering and regenerative medicine (Kfoury and Scadden, 2015). Furthermore, emerging experimentation elucidating immunomodulation, tissue regeneration, anti-aging ability and *in vivo* biology of MSCs has prompted their potent applications in cell-based therapy (Yue et al., 2016; Carr et al., 2019; Fu et al., 2019; Yang et al., 2019). Recent experiments with MSCs have been applied to the study and treatment of COVID-19, and the rapid response to emergent diseases is evidence of the promise of MSCs in the treatment of immune and infectious diseases (Li et al., 2020; Meng et al., 2020; Abdelgawad et al., 2021; Shi L. et al., 2021).

### 2.1 Functional characteristics of MSCs

According to this definition, the basic functional characteristics of MSCs are plastic adherent and clonogenic *ex vivo*, by which MSCs can be isolated. MSCs induced in conditioned medium for a period of time can be stained with alkaline phosphatase and alizarin red *in vitro* and express a series of markers of osteogenic differentiation such as alkaline phosphatase (ALP), Runt-related transcription factor 2 (RUNX2), osteocalcin (OCN) and osterix (OSX). In addition, MSCs induced in conditioned medium can be stained with oil red O and microscopically show obvious lipid droplet formation and express markers of lipogenic differentiation such as peroxisome proliferator activated receptor gamma (PPARγ) and lipoprotein lipase (LPL). The self-renewal and multipotency of MSCs were further confirmed *in vivo* though serial transplantation assays, with labeling of green fluorescent protein (GFP) or surface markers such as Nestin and CD146 , demonstrating the ability to reconstitute heterotopic ossicles when implanted subcutaneously and maintain identical phenotypes when generating secondary MSCs and ossicles. In addition to self-renewal and multipotency, MSCs have been identified as potent immunosuppressors (Bárcia et al., 2015; Yang et al., 2017; Vázquez et al., 2020). They possess the ability to modulate innate immune responses (Jiang et al., 2021; Dave et al., 2022), suppress the proliferation and differentiation of B cells, induce T-cell apoptosis and restore the balance between T-cell subsets, and rescue the onset of inflammation. The immunomodulatory/anti-inflammatory capacity of MSCs has been proven to be particularly important when MSCs treating autoimmune or skeletal degenerative diseases (Csobonyeiova et al., 2017; Jiang et al., 2021; Zhu et al., 2021) upon systemic delivery. Another capacity of MSCs is migration, which enable them to migrate toward damaged tissues during development and after systemic infusion, to differentiate into functional cells that exert a reparative therapeutic effect, or to fuse with cells at the site of damage and then regenerate the damaged tissue (Chen et al., 2016; Liesveld et al., 2020; Wang et al., 2020; Galgaro et al., 2021; Lee et al., 2021; Ma et al., 2021). All these repair processes suggest that MSCs can be mobilized to functional sites for endogenous tissue regeneration and functional remodeling. Recent studies have also shown that MSCs exert therapeutic effects through paracrine effects, such as miRNAs, cytokines and chemokines, which can improve the pathological microenvironment and repair locally damaged tissues; and that mitochondrial transfer mechanisms can provide functional recovery after the repair of mitochondrial dysfunction caused by aging (Babenko et al., 2018). MSCs-derived extracellular vesicles (EVs) have been the focus of recent research, producing membrane-enclosed vesicles in response to external stimuli and playing a critical role in regulating the immune microenvironment, inhibiting inflammatory factor expression and promoting angiogenesis (Babenko et al., 2018).

### 2.2 Dynamic interactions with microenvironments

A conspicuous functional characteristic of MSCs is their reciprocal regulation with the surrounding niche/microenvironment. MSCs reside in a complex architecture composed of neighboring cells and abundant neurovascular bundles (Zhao et al., 2018; Imhof et al., 2020; Wu et al., 2022). MSC behaviors of quiescence and activation of MSCs are tightly controlled by the local niche according to the requirements of the host tissues (Baccin et al., 2020; Hageman et al., 2020). MSCs also accept long-distance regulation by the circulatory microenvironment through soluble factors, such as hormones (for example, estrogen), metabolites (for example, glucose) and inflammatory cytokines (for example, tumor necrosis factor-alpha [TNF-α] and interferon-gamma [IFN-γ]). (Sala et al., 2015; Sui B. D. et al., 2016; Simovic Markovic et al., 2017). MSCs modulate the ambient microenvironmental properties through cell-cell contact and paracrine secretion of various cytokines (Li et al., 2019; Song et al., 2020) and EVs (Liu S. et al., 2015; Hade et al., 2021). MSCs can produce gasotransmitters of nitric oxide (NO) (Ren et al., 2008) and hydrogen sulfide (H_2_S) (Liu et al., 2014) to create favorable microenvironments through autocrine/paracrine regulatory loops. These mutual communications between MSCs and microenvironments, particularly those connecting MSCs with the host immune systems and release-based interactions, provides crucial mechanisms underlying the therapeutic applications of MSCs (Ansari et al., 2017; Weiss et al., 2019; Planat-Benard et al., 2021; Yao et al., 2021).

## 3 Roles of MSC in organismal homeostasis and disease

Despite the well-documented experimentation describing the functional characteristics of MSCs, including easy access, anti-inflammatory activity mainly in the form of a thirst for damaged cells, immune modulation and regeneration promotion, a fundamental issue concerns the identity and physiological function *in vivo* (**Figure 1**). However, studies in this field have been hampered by a lack of MSC-specific antigens that permit both prospective identification and fate mapping. According to the latest statistic, it is estimated that > 9233 clinical trials have been registered and conducted on MSCs, but the lack of adequate standardized methods to assess the main safety issues involved in MSCs, specifically the lack of MSC-specific antigens for prospective identification and fate localization, has hindered their use in clinical settings. Until recently, lineage tracing and ablation studies identified several surface markers representing subsets of MSCs related to their respective nature and niches. In particular, regarding the ability of MSCs to trigger and promote tumorigenesis, although few studies have supported that MSCs are relatively safe for clinical use, the same MSCs applied to other receptors or tissues and organs also require further discussion and study.

The developmental origin and function of MSCs remain an active area of research. Although it was originally believed that MSCs (particularly BMMSCs) are derived from the mesoderm and give rise to mesenchymal cells, such as osteoblasts, adipocytes and chondrocytes (Dennis and Charbord, 2002), evidence has emerged that suggests the existence of different MSC subsets during development and possibly in adults with distinct origins and functions. Dental MSCs are generated from a unique neural crest or glial source in development (Miletich and Sharpe, 2004; Kaukua et al., 2014); most recently, Nestin^+^ BMMSCs have also been revealed as descendants of the neural crest, and unlike mesoderm-derived MSCs, they maintain hematopoietic stem cells (HSCs) but do not contribute to fetal osteochondrogenesis (Isern et al., 2014). Therefore, as an HSC niche component, MSCs have been detected in major hematopoietic sites during mouse development, such as the aorta-gonad-mesonephros and fetal liver during mid-gestation and in neonatal and adult BM (Mendes et al., 2005). However, the developmental hematopoietic function of MSCs seems to depend on their bone-forming capacity in the BM, where Osx-deficient MSCs fail to form osteoblasts in the metaphyseal area with reduced HSC function (Coşkun et al., 2014). Despite these findings, whether fetal MSCs function differently than postnatal MSCs remain unclear.

The postnatal roles of MSCs in tissue homeostasis is clearly understood. In this regard, BMMSCs have been intensively investigated for their putative contribution to skeletal remodeling (Chan et al., 2015; Worthley et al., 2015) and hematopoiesis (Derecka et al., 2020; Borella et al., 2021; Cai et al., 2022; Schloss et al., 2022). For instance, leptin receptor (Lepr)^+^ (Yue et al., 2016; Schloss et al., 2022) and Gremlin1^+^ (Worthley et al., 2015) cells are enriched for osteogenesis with either adipogenesis or chondrogenesis of perivascular BMMSCs in the adult skeletal system, and Nestin^+^ (Burt et al., 2019; Nobre et al., 2021) and platelet-derived growth factor receptor alpha (PDGFRα)^+^CD51^+^ (Lawal et al., 2017; Rux et al., 2017; Mennan et al., 2019) cells have shown co-segregation between colony-forming activity and HSC maintenance activity of BMMSCs. Moreover, dental MSCs have been shown to actively participate in the dynamic turnover of craniofacial bone (Zhao et al., 2015) and dental tissues, as represented by Gli1^+^ MSC subsets. However, critical questions remain as to whether different MSC markers overlap with each other and how distinct MSC subsets coordinate tissue homeostasis and diseases.

Although specific markers with related physiological function of MSCs *in vivo* remain to be elucidated, their pathophysiological contributions to diseases, as shown by declined or altered behaviors in situ and *ex vivo*, have been recognized in skeletal and dental systems. Osteoporosis, the skeletal degenerative disease, is characterized by loss of bone mass with increased marrow adiposity. It has been well documented that BMMSCs suffer from reduced proliferation with a differentiation shift from osteogenesis to adipogenesis in osteopenias of diverse pathologies (Liu et al., 2016; Sui et al., 2016a; Li et al., 2017). These functional impairments of BMMSCs could be attributed to the detrimental effects of diseased microenvironmental factors, such as estrogen deficiency and inflammation status (Chen et al., 2015; Shao et al., 2015). Similar damages to local resident MSCs have also been observed in other inflammatory conditions, e.g. osteoarthritis (Zhen et al., 2013) and periodontitis (Xue et al., 2016). Besides, ablation of Gli1+ cells leads to craniosynostosis and arrest of skull growth, indicating MSCs are indeed indispensible for skeletal homeostasis (Zhao et al., 2015). In addition, MSC aberrations have been revealed as a key pathogenesis in mutant-HSC-driven leukemia, in which BMMSCs could be impaired by neuropathy of the marrow niche to alter their HSC-maintaining secretome (Dong et al., 2016). The above findings further confirm the pathophysiological importance of MSC interactions with microenvironments in tissue homeostasis and diseases.

### 3.1 *In situ* regeneration: Stepping toward a future therapeutic option

Given the putative key roles of MSCs in tissue homeostasis, a strategy of *in situ* regeneration has been proposed to reverse the functional decline of resident MSCs in treating degenerative diseases. Through inhibition of microenvironmental inflammatory impacts, systemically infusion of neutralizing antibodies of either TNF-α or IFN-γ, as well as the non-steroidal anti-inflammatory drug aspirin, has been documented to be sufficient to rescue BMMSC deficiency in osteoporosis (Sala et al., 2015; Liao et al., 2016; Xu et al., 2016; Lu et al., 2017; Simovic Markovic et al., 2017; Chang et al., 2022). Microenvironmental agents for the improvement of MSC function in osteoporosis have also been reported to include the gasotransmitter H_2_S donor GYY4317 and Insulin-like growth factor 1 (IGF1), together with its binding protein (IGFBP3). More agents have been developed based on mechanistic studies unraveling pharmacological targets in MSC functional regulation, such as the mammalian target of rapamycin (mTOR) signaling inhibitor rapamycin (Chen et al., 2015; Liu et al., 2016), the Notch signaling inhibitor DAPT (Liu S. et al., 2015), the nuclear transcription factor-kappa B (NF-κB) signaling inhibitor PDTC, and the migration stimulator LLP2A-Ale for directing MSCs to bone formation surfaces.

Despite the fact that these pharmacological interventions have proven effective in restoring MSC function in certain disease models, *in situ* MSC-based regeneration is still in the process of becoming a feasible option, as limitations still exist, including the specificity and sustainability of their therapeutic influences. To date, no MSC-targeted agents have been approved or applied in clinically. Current preclinical and clinical studies have more extensively applied exogenous MSC cytotherapy in harnessing MSCs for therapeutic use, which has been shown to be capable of restoring endogenous MSC function (Liu S. et al., 2015; Chen et al., 2017), as discussed below.

## 4 MSC therapy: The efficacy based on cell-host interplay

**Figure 2:**
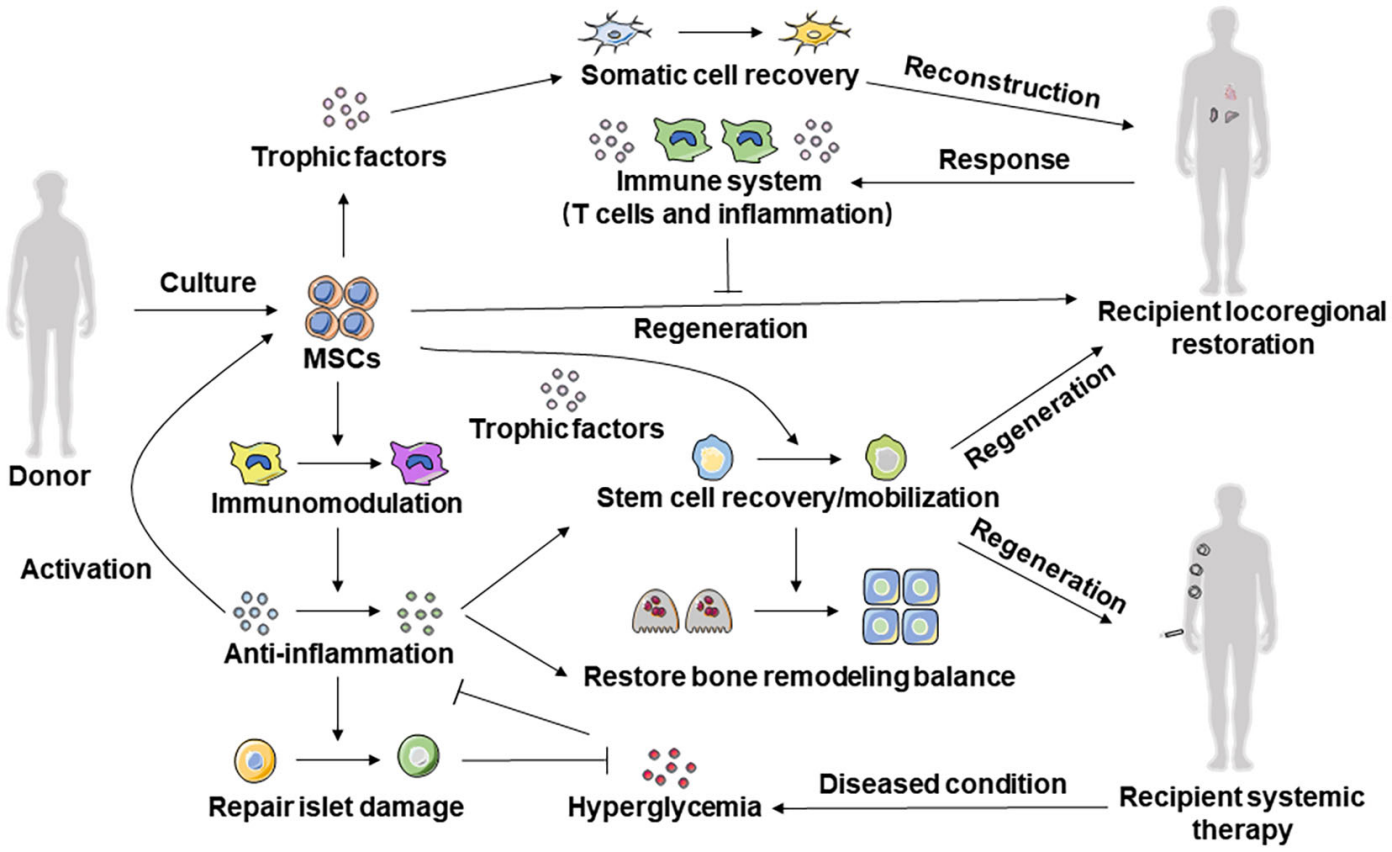
Cell-host interplay determines efficacy of MSC therapy. Systemic infusion and local transplantation of MSCs can secrete anti-inflammatory factors to target cells through immunomodulatory ability to repair damaged tissues and maintain local tissue immune homeostasis. A variety of cytokines and EV forms of tissue trophic factors can be secreted through the paracrine effect to promote mobilization, proliferation, and anti-apoptosis, to improve the microenvironment of the recipient and inhibit the development and progression of disease.

The functional characteristics of MSCs indicate their therapeutic potential. In support of this, research on MSC therapy has experienced unprecedented advances in recent years, becoming the focus of extensive work worldwide to develop approaches for a variety of diseases. In particular, evidence for the potent efficacy of MSC therapy comes from in-depth understanding of the restoration and mechanisms of systemically delivered MSCs in recipient tissue, microenvironmental homeostasis, and endogenous stem cell function. Interestingly, the benefits of MSCs are wide-ranging and remain detectable long after the disappearance of transplanted MSCs (Liu S. et al., 2015; Ng et al., 2015). Accordingly, the current recognition of MSC therapy has advanced from cell-autonomous functional determination to the essence of cell-host interplay (**Figure 2**).

The landscape of current MSC therapy contains at least two aspects: systemic and locoregional delivery of exogenous MSCs. Among these two application strategies, systemic MSC cytotherapy, primarily through intravenous and intraperitoneal injections, is currently a research hotspot in stem cell therapy, which has been recognized to have therapeutic effects in various diseases. In particular, the immunomodulatory capability of MSCs has made them attractive and potent candidates for autoimmune and inflammatory conditions, in which they can modify the systemic microenvironment further toward a beneficial environment for tissue repair (Shi et al., 2018; Medhat et al., 2019; Borella et al., 2021; Markov et al., 2021). Their ability to suppress immune responses has become the basis for numerous preclinical and clinical studies on a range of systemic conditions and their complications, including systemic lupus erythematosus (SLE), graft versus host disease (GvHD), rheumatoid arthritis (RA) (Liu R. et al., 2015; Gu and Shi, 2016), systemic sclerosis (SSc), inflammatory bowel disease (IBD), type 1 and type 2 diabetes (T1D and T2D), osteoporosis, and osteonecrosis. Furthermore, systemically delivered MSCs benefit locoregional lesions in diverse tissues, such as myocardial infarction (Luo et al., 2017), liver fibrosis, (Watanabe et al., 2019; Shi M. et al., 2021) and renal failure (Yun and Lee, 2019). The promise of MSC cytotherapy in restoring organismal homeostasis has ushered in hundreds of clinical trials that have employed systemic infusion of MSCs.

Locoregional application, has been extensively investigated in regenerative medicine. Studies based on tissue engineering techniques have successfully regenerated/repaired craniofacial (Yu et al., 2021) and long bone defects (Zhao et al., 2021), cartilage (Kangari et al., 2020), functional tooth roots and dental pulp (Xuan et al., 2018; Guo et al., 2021), periodontal structures (Sui et al., 2019), cutaneous wounds (An et al., 2015), infarcted myocardium, neurons and nerves. Furthermore, applications are in continuous progress by optimizing MSC viability with the cell-sheet/cell-aggregate technique (Xuan et al., 2018), improving scaffold materials with nanotechnology (Kuang et al., 2016) or microencapsulation (Moshaverinia et al., 2015), and combining favorable agents for MSC function with preconditioning (Shuai et al., 2016) or co-delivery systems (Moshaverinia et al., 2015). However, a significant challenge in this field is to maintain the viability of implanted MSCs in diseased microenvironments and to maximize their efficacy during the survival period. It has been reported that recipient immune systems, primarily T cells and secreted TNF-α and IFN-γ, remarkably inhibit MSC-mediated tissue regeneration by inducing MSC apoptosis and impairing MSC differentiation (López-García and Castro-Manrreza, 2021; Xie et al., 2021) (**Figure 2**). Further diminishing the regenerative potential, the detrimental impacts of donor comorbidities regarding aging, inflammation, and hyperglycemia are just beginning to emerge, constituting the main barrier for the application of autologous MSCs. Therefore, further studies are required toward optimizing locoregional application of MSCs, compared to systemic MSC therapy, which demonstrates distinctive advantages of minimum injuries and stable, long-lasting, and wide-ranging beneficial effects.

## 5 Cell-host interplay in systemic MSC therapy

### 5.1 Long-term restoration of recipient homeostasis

One of the most profound cell-host interplay in MSC therapy is the re-establishment of the recipient immunological balance, particularly homeostasis among T-cell subsets. Generally, systemic MSC infusion can reduce the number of CD3^+^ T cells by inhibiting proliferation and inducing apoptosis (Fujii et al., 2018; Sui et al., 2018) while also reducing recipient CD4^+^ and CD8^+^ T-cell populations (Wang et al., 2018; Harrell et al., 2021). Specifically, for the CD4^+^ T-cell subsets, the infused MSCs can suppress pro-inflammatory T helper 1 (Th1) and Th17 cells while promoting anti-inflammatory Th2 and CD4^+^CD25^+^Foxp3^+^ regulatory T cells (Tregs), thus restoring the functional balance (Court et al., 2020). Mechanistically, the infused MSCs exert combined effects by paracrine secretion and cell-cell contact: MSCs secrete monocyte chemotactic protein 1 (MCP-1) to recruit T cells (Liu et al., 2021) and various immunosuppressive cytokines such as NO and indoleamine 2,3-dioxygenase (IDO), and they express FAS ligand (FASL) and bind to FAS on T-cell surfaces to induce apoptosis (Liu et al., 2021). Interestingly, MSCs also express FAS, which plays an important role in regulating MCP-1 secretion to enhance recruitment. Apoptotic T cells can be further engulfed by macrophages and can stimulate macrophage TGF-β production to induce Tregs for immune tolerance. Surprisingly, these effects start as early as 1.5 h post-infusion in mice and last as long as 12–18 months in humans post-infusion. The amazingly persistent efficacy indicates unrecognized mechanisms underlying the long-term functional restoration of each T-cell subset. Similar phenomena were also detected when MSCs maintained macrophage homeostasis by promoting anti-inflammatory M2 polarization and inhibiting pro-inflammatory M1 polarization (Lee et al., 2015; Xie et al., 2016; Pajarinen et al., 2019; Arabpour et al., 2021).

Another example of MSC re-establishment of functional homeostasis is the restored bone remodeling balance observed in systemic MSC therapy. In osteoporotic cytotherapy, the systemic infusion of MSCs rescued the impaired bone formation rate and reduced the stimulated bone resorption rate under diverse pathological conditions (Liu S. et al., 2015; Chen et al., 2017; Sui et al., 2017). The efficacy can be attributed to increased osteoblastogenesis with decreased osteoclastogenesis through indirect mechanisms through immunomodulation (Sui et al., 2017), secretion (Liu S. et al., 2015; Chen et al., 2017), or potential homing (Sui et al., 2016b). Interestingly, skeletal therapeutic effects are also long-lasting (at least 8–12 weeks) (Liu S. et al., 2015; Chen et al., 2017; Sui et al., 2017). The paracrine effects of transplanted MSCs on other types of recipient somatic cells, including cardiac myocytes (Cheng et al., 2020), epithelial cells (Nagaishi et al., 2016), endothelial cells (Lin et al., 2015), fibroblasts (Picke et al., 2018), smooth muscle cells (Cheng et al., 2017), adipocytes (Xie et al., 2016), and neurons (He et al., 2021), have also been reported during MSC application in myocardial infarction, nephropathy, atherosclerosis, scar formation, pulmonary hypertension, insulin resistance, and axon guidance. Collectively, these findings reveal the critical role of cell-host interplay in mediating the wide-ranging and long-lasting efficacy of MSC therapy.

In addition to the functional restorations of recipient cellular components, both systemic and local microenvironments can be modified by MSC therapy toward beneficial circumstances for tissue repair. Depending on their immunomodulatory capacity, infused MSCs exert potent anti-inflammatory effects in the circulation and tissue niches, underlying indirect therapeutic efficacy in inflammation-induced bone and pancreatic islet defects (Jin et al., 2019; Gan et al., 2020; Zhou et al., 2020). The regenerated islet β cells lead to the secondary rescue of hyperglycemia, which is beneficial for addressing diabetic complications. Furthermore, both systemically infused and locoregionally transplanted MSCs secrete numerous tissue trophic factors in the form of cytokines and EVs, which possess various microenvironment-improving effects such as pro-mobilization, pro-proliferation (Deng et al., 2016; Grange et al., 2019; Mathew et al., 2019), anti-apoptosis (Nagaishi et al., 2016; Grange et al., 2019), and pro-/anti-angiogenesis (Todeschi et al., 2015; Zanotti et al., 2016; Xiao et al., 2021), indicating general recovery of diseased recipient microenvironments (**Figure 2**).

Notably, cell-host interplay indicates reciprocal interactions in that recipient microenvironmental status also greatly influences the therapeutic performance of MSCs. In addition to the pro-inflammatory T cells in recipients that inhibit MSC regeneration through synergistic effects of IFN-γ and TNF-α, crosstalk between transplanted donor MSCs and the recipient immune system also exists in the systemic application of MSCs, but functions distinctively to trigger immunomodulation of exogenous MSCs. In particular, recipient IFN-γ combined with other pro-inflammatory cytokines induce the secretion of chemokines and NO by exogenous MSCs, which recruit and inhibit recipient T cells, respectively. Recipient IFN-γ also elicits the expression and secretion of other immunoregulatory cytokines of donor MSCs such as IDO, thus rescuing the impaired immunosuppressive function of diseased MSCs. Another recipient factor, nevertheless, participates as a regulatory element in the immunosuppressive function, in that the recipient hyperglycemic microenvironment diminishes immunomodulation and therapeutic effects of systemically infused MSCs on osteopenia (Le Blanc et al., 2004; Sui et al., 2017). These findings integrate a previously unrecognized axis into the cell-host interplay in MSC therapy and ravel that the fulfillment of potent therapeutic effects of MSCs requires critical assistance from and a high level of control of recipient microenvironments.

### 5.2 Persistent rescue of endogenous stem cell deficiency

These findings, particularly the long-term restoration of recipient homeostasis, prompt further investigations on the functional recovery of recipient resident stem cells. Because of the increased osteoblastogenesis observed in MSC treating osteoporosis, osteogenic differentiation of recipient BMMSCs has been extensively examined. As expected, rescue of impaired osteogenesis of recipient BMMSCs has been detected in MSC therapies in various murine models, including osteopenia induced by estrogen deficiency (ovariectomy [OVX]), SLE (Liu S. et al., 2015; Ma et al., 2015), and SSc (Chen et al., 2017). Furthermore, recipient BMMSCs exhibited enhanced bone regenerative capability when transplanted ectopically, suggesting correlations with restored bone formation rates *in situ* (Liu S. et al., 2015; Chen et al., 2017). Recipient BMMSCs also showed stimulated colony-forming capacity after allogeneic MSC infusion (Chen et al., 2017). In addition, exogenous MSC therapy inhibits adipogenesis (Chen et al., 2017) and osteoclastic induction (Ma et al., 2015) of resident MSCs, thereby restoring skeletal homeostasis. Importantly, the functional recovery of recipient BMMSCs persisted for at least 12 weeks post-infusion (Liu S. et al., 2015), again indicating that a single administration of MSCs is capable of maintaining the therapeutic effects for a sustained period of time.

The effects of MSC therapy on the stimulate function of recipient endogenous stem cells have been observed in other systems. Intramuscular injection of prostacyclin-overexpressing MSCs promoted the survival and proliferation of host muscle progenitor cells under hypoxic conditions to show enhanced muscle regeneration in a murine hindlimb ischemia model (Deng et al., 2016). Systemic MSC therapy may also improve pancreatic islet β-cell regeneration to increase insulin production in T1D mice. The subcutaneously transplanted MSCs show long-distance chemotactic and inductive activity on recipient HSCs to form analog BM elements with ectopic hematopoiesis, which can rescue lethally irradiated mice and alleviate aging-related phenotypes in immunocompromised mice. These functional recoveries of endogenous stem cells, together with those observed in BMMSCs, are primarily attributed to the paracrine effects of donor MSCs, rather than their prolonged engraftment in recipient tissues (Liu S. et al., 2015; Deng et al., 2016; Rahmani et al., 2020; Ma et al., 2021; Xiao et al., 2021).

In summary, the above result revealed the extensive efficacy of MSC therapy based on cell-host interplay to trigger intensive restoration of recipient function (**Figure 2**). These effects, particularly the persistent functional recovery of recipient cells observed in systemic MSC therapy, suggest the existence of critical molecular alterations that mediate the long-term detectable therapeutic benefits.

## 6 Conclusions and future perspectives

The promise of stem cell therapy in regenerating damaged tissues and restoring organismal homeostasis in aging and diseases has prompted thousands of clinical trials including > 700 that employ MSCs. This relies on critical molecular mechanisms. Moreover, in the future, specific interaction mechanisms will be based on the paracrine mode of action, and the study of the effects of EVs will become a hot topic in this field.

MSC therapy is a hot topic based on its current translational application and MSCs have recently been listed as promising drugs for the treatment of COVID-19, thus demonstrating their important role in the treatment of inflammatory and immune diseases. However, the feasibility and safety of MSCs have only been tested, and there is a lack of sufficient evidence on their therapeutic efficacy, particularly with regard to the lack of clear evidence to fully characterize their potential therapeutic sequelae. Although some MSCs have been shown to be safe and effective for clinical use, it is uncertain whether this can be extended to other tissues. Their therapeutic risks are mainly focused on their heterogeneity and on the initiation and promotion of tumor production; however exosomes have been shown to circumvent these concerns and are safer to use in clinical regeneration. In conclusion, extensive study is still required before MSCs can be used in a manner and extended to wider clinical applications.

## Author contributions

PL designed, drafted and revised the manuscript. YA, TZ critically contributed to the design of the paper for important intellectual content. ST, XH, SL and FF substantially contributed to the conception of the study. JC, KX conceived and supervised the study and revised the paper. All authors contributed to the article and approved the submitted version.
